# West Nile virus neuroinvasive disease and cardiac involvement in critically ill patients in central Italy: a case series

**DOI:** 10.3389/fmed.2026.1792053

**Published:** 2026-03-13

**Authors:** Nardi Tetaj, Maria Grazia Bocci, Giulia Capecchi, Antonio Lesci, Dorotea Rubino, Valerio Sabatini, Giorgia Taloni, Elena Mattiucci, Tommaso Ascoli, Gaetano Maffongelli, Patrizia De Marco, Francesca Moschella Orsini, Simone Burocchi, Fabrizio Albarello, Stefania Ianniello, Alberta Villanacci, Martina Nocioni, Cosmo Del Borgo, Fabio Alfredo Nania, Nicola Petrosillo, Francesco Vairo, Emanuele Nicastri

**Affiliations:** 1 Cardiology Unit, Fondazione Policlinico Universitario Campus Bio-Medico, Rome, Italy; 2 Department of Clinical and Clinical Research, National Institute for Infectious Disease “Lazzaro Spallanzani”—IRCCS, Rome, Italy; 3 Istituto di Anestesiologia e Rianimazione, Università Cattolica del Sacro Cuore, Rome, Italy; 4 Clinical Division of Infectious Diseases, National Institute for Infectious Disease “Lazzaro Spallanzani”—IRCCS, Rome, Italy; 5 Cardiology Unit, National Institute for Infectious Disease “Lazzaro Spallanzani”—IRCCS, Rome, Italy; 6 Radiology Unit, National Institute for Infectious Diseases “Lazzaro Spallanzani”—IRCCS, Rome, Italy; 7 Department of Anesthesia and Intensive Care Unit, Santa Maria Goretti Hospital, Latina, Italy; 8 Fondazione Policlinico Universitario Campus Bio-Medico, Rome, Italy; 9 Regional Service for Surveillance and Control of Infectious Diseases (SERESMI) Lazio Region, National Institute for Infectious Diseases “Lazzaro Spallanzani”—IRCCS, Rome, Italy

**Keywords:** cardiac involvement, critical care, encephalitis, intensive care unit, myocarditis, neuroinvasive disease, West Nile virus

## Abstract

**Introduction:**

West Nile virus (WNV) infection represents an emerging health concern in Southern Europe. Although most infections are asymptomatic, a small proportion progress to WNV neuroinvasive disease (WNND), occasionally complicated by cardiac involvement. Data describing critically ill patients with WNND and cardiac involvement requiring intensive care (ICU) are limited.

**Methods:**

We conducted a two-month observational case-series of consecutive adult patients with confirmed WNND admitted to the ICUs of two tertiary hospitals in Lazio region, Italy from July 1 to September 1, 2025. Clinical, laboratory, imaging, and outcome data were collected, with particular focus on neurological and cardiac manifestations.

**Results:**

Among 197 patients with confirmed WNV infection, 64 (32%) had WNND, of whom 12 (6.1%) required ICU admission. These 12 patients were subsequently enrolled in the study. The median age was 76.5 years (IQR 70–83), and 75% were male. The most common symptoms were fever, myalgia, and gastrointestinal complaints, followed by confusion, limb weakness, and psychomotor slowing. Cerebrospinal fluid analysis showed lymphocytic pleocytosis (median 135 cells/mm^3^), elevated protein (median 126 mg/dL), and normal glucose. Brain MRI revealed T2/FLAIR hyperintensities in the thalami and brainstem in most cases, 2 patients had normal imaging. Four patients had cardiac involvement; two fulfilled criteria for suspected myocarditis and one was diagnosed with Takotsubo syndrome, based on new left ventricular dysfunction, elevated troponin, and diffuse hypokinesis not confined to a coronary territory. Five patients (41.7%) died within 30 days of admission. Non-survivors were older (median 83 years) and had more comorbidities. Survivors showed higher inflammatory markers.

**Conclusion:**

WNND remains a life-threatening condition requiring multi-disciplinary critical care. Cardiac involvement, including suspected myocarditis, was observed in a substantial proportion of critically ill WNND patients and occurred more frequently among non-survivors in this cohort. Although descriptive, these findings highlight the importance of early neuro-cardiac assessment and comprehensive supportive management to optimize outcomes in severe WNND.

## Introduction

West Nile Virus (WNV) is a single-stranded positive-sense RNA virus, maintained in an enzootic cycle between migratory birds and Culex mosquitoes, while humans are considered dead-end hosts (1). Following inoculation via the saliva of an infected mosquito, WNV initially migrates to regional draining lymph nodes, where viral replication occurs before dissemination into the bloodstream and subsequent spread to visceral organs.

WNV was first isolated from a patient from the West Nile subregion of Uganda in 1937, and only in the late 1990s began to emerge as a cause of severe neurologic disease in North America and Europe, causing recurrent seasonal outbreaks with significant public health impact (2). The global dissemination of WNV has been partly driven by climatic and environmental changes, which have favored the expansion of mosquito habitats and altered migratory patterns of reservoir hosts (3). These factors have facilitated the geographic spread of the virus and contributed to the growing burden of infection, now affecting millions of individuals worldwide (4–6). In Italy, sporadic cases have been reported since 2008, with an increasing number of outbreaks in recent years, particularly in the northern regions (7, 8). During summer 2025 an outbreak of WNV was identified for the first time in the Lazio Region, central Italy. As of August 18, 171 autochthonous cases with WNV infection were identified with 57 (33.3%) cases of neuroinvasive syndrome, underscoring the clinical relevance of severe presentations in this region (9).

In susceptible individuals, the virus may cross the blood–brain barrier and invade the central nervous system (CNS) (10, 11). Fewer than 1% of WNV infections progress to neuroinvasive disease, manifesting as meningitis, encephalitis, acute flaccid paralysis, or Guillan-Barré syndrome (12, 13). Long-term neurologic sequelae are common among survivors (14). Cardiac involvement represents a rare but potentially life-threatening complication, often underrecognized due to its nonspecific clinical presentation and overlap with other critical conditions (15, 16).

This case series aims to provide a comprehensive description of the clinical characteristics, diagnostic evaluation, management strategies, and outcomes in patients with WNND, with particular emphasis on cardiac involvement of the cases that required an ICU admission.

## Materials and methods

### Study design and setting

We conducted a multicenter observational study of critically ill patients with WNND admitted to ICU of two tertiary care hospitals in central Italy during 2 months of 2025 outbreak (from July 1 to September 1, 2025), at the National Institute for Infection Diseases Lazzaro Spallanzani, Rome, and Santa Maria Goretti Hospital, Latina.

### Patient selection and diagnosis

Eligible participants were adults (≥18 years) admitted to the ICU of either center with confirmed WNV infection, defined by the presence of WNV-specific IgM antibodies in serum or cerebrospinal fluid (CSF) detected by enzyme-linked immunosorbent assay (ELISA), or by a positive molecular test (RT-PCR) in blood, CSF, or tissue samples. Virus neutralization tests were not routinely performed due to laboratory workflow constraints during the outbreak. However, cases were managed in referral infectious disease centers following regional surveillance protocols.

CSF profile data were collected from the first lumbar puncture, whether performed at a referring hospital or upon admission to the participating institutions. Imaging findings were based on the first available MRI with a complete report, and all images were reviewed by a board-certified neuroradiologist. Electromyography (EMG) results were extracted from the full reports provided by a board-certified neurophysiologist.

The case definition of neuroinvasive disease required the presence of encephalopathy (depressed or altered mental status, lethargy, or personality changes) together with at least two of the following criteria: fever (≥38 °C) or hypothermia (≤35 °C); cerebrospinal fluid (CSF) pleocytosis (≥5 cells/mm^3^); neuroimaging findings consistent with encephalitis; focal neurological deficits; meningism; electroencephalography (EEG) abnormalities compatible with encephalitis; or seizures (17). Encephalitis was defined as altered mental status (e.g., confusion, lethargy, personality change, or decreased level of consciousness) lasting ≥24 h, accompanied by at least one supportive feature including cerebrospinal fluid (CSF) pleocytosis, compatible neuroimaging abnormalities, electroencephalographic changes, or seizures. Meningitis was defined as the presence of meningeal signs (e.g., headache, neck stiffness, photophobia) with CSF pleocytosis and virological confirmation of WNV, in the absence of significant parenchymal brain dysfunction. Acute flaccid paralysis was defined as acute onset limb weakness with lower motor neuron signs, with or without supportive electromyography findings, in patients with laboratory-confirmed WNV infection, after exclusion of alternative etiologies.

Cardiac involvement was defined as the occurrence during hospitalization of new-onset myocardial injury or dysfunction temporally associated with WNV infection and not fully explained by pre-existing cardiac disease. Operationally, this required at least one of the following: (i) elevated cardiac biomarkers (troponin with or without NT-proBNP increase), (ii) new electrocardiographic abnormalities (ST-segment changes, T-wave inversion, arrhythmias), and/or (iii) new left ventricular systolic dysfunction or regional wall motion abnormalities on transthoracic echocardiography. Suspected myocarditis was diagnosed in the presence of dynamic troponin elevation and new ventricular dysfunction not confined to a single coronary territory, in the absence of angiographic evidence of acute culprit coronary stenosis when coronary angiography was performed, consistent with contemporary ESC recommendations. Takotsubo syndrome was defined by transient left ventricular systolic dysfunction with a typical apical or mid-ventricular ballooning pattern extending beyond a single epicardial vascular distribution, associated with modest biomarker elevation and absence of obstructive coronary disease when evaluated. Definitive myocarditis confirmation by cardiac magnetic resonance (CMR) or endomyocardial biopsy (EMB) was not feasible in most critically ill patients; therefore, diagnoses were considered probable based on clinical and instrumental criteria (17, 18). Among consecutive adult patients diagnosed with WNND during the study period, all patients requiring ICU admission for severe neurological impairment, respiratory failure, hemodynamic instability, or other life-threatening complications were included in the present case series.

### Data collection

Collected data included demographic variables (age, sex, body mass index or BMI), initial symptoms, and comorbidities such as hypertension, coronary artery disease, atrial fibrillation, diabetes, obesity (BMI > 30 kg/m^2^), chronic renal or liver disease, chronic pulmonary disease (including COPD), chronic neurological disorders, solid or hematological malignancies, autoimmune disease, organ transplantation, and other chronic conditions.

During hospitalization, we also recorded pre-ICU hospitalization days, the occurrence and timing of endotracheal intubation, extubation procedures, and outcomes (discharge or death).

All clinical management decisions were made by attending physicians in accordance with published guidelines and standard medical practice. Data collection followed a standardized protocol to minimize recording bias. The primary outcome was 30-day mortality, selected as a standardized and clinically relevant endpoint commonly used in critical care infectious disease studies. ICU mortality and in-hospital mortality were also assessed.

### Ethical approval

The study was approved by the Institutional Review Board (Comitato Etico Territoriale Lazio Area 4) of the National Institute for Infectious Disease- L. Spallanzani (approval code, 76–2025). The study was conducted according to the guidelines of the Declaration of Helsinki.

Due to its retrospective nature, informed consent was waived.

## Results

During the study period, a total of 197 patients were virologically confirmed WNV infection in the two participating tertiary centers. Among them, 64 (32%) had a definitive diagnosis of WNV neuroinvasive disease (WNND), of which 12 patients requiring ICU admission were included between July 1 and September 1, 2025. No ICU cases were excluded, and no early deaths or inter-hospital transfers occurred prior to ICU assessment. Therefore, the cohort represents all severe WNND cases requiring intensive care management during the study period. ICU mortality and in-hospital mortality were identical to 30-day mortality in this cohort, as no additional deaths occurred after hospital discharge within the 30-day follow-up period. The most common initial symptoms were fever (100%), fatigue (67%), myalgia (67%), and gastrointestinal manifestations (50%), including nausea, vomiting, and diarrhea, followed during disease progression by neurological symptoms such as headache, confusion, photophobia, and limb weakness (Table 1).

**Table 1:** Baseline symptoms of critically ill WNV patients.

Symptoms	Total (%)	Survivor group	Non-survivor group
Patients number	12	7 (58%)	5 (42%)
Fever	12 (100%)	7 (100%)	5 (100%)
Gastrointestinal symptoms	6 (50%)	4 (57%)	2 (40%)
Fatigue	8 (66.7%)	5 (71.4%)	3 (60%)
Myalgia	8 (66.7%)	5 (71.4%)	3 (60%)
Skin rash	2 (16.7%)	1 (14.2%)	1 (20%)
Headache	8 (66.7%)	5 (71.4%)	3 (60%)
Photophobia	3 (25%)	2 (28.5%)	1 (20%)
Limb weakness	3 (25%)	2 (28.5%)	1 (20%)
Stiff neck	2 (16.7%)	1 (14.2%)	1 (20%)
Diplopia	2 (16.7%)	2 (28.5%)	0 (0%)
Dysarthria	3 (25%)	2 (28.5%)	1 (20%)
Confusion	7 (58.3%)	4 (57%)	3 (60%)
Focal neurological deficit	1 (8.3%)	0 (0%)	1 (20%)

Nine patients (75%) were male, with a median age of 76.5 years (IQR 69.3–83). The most prevalent comorbidities were arterial hypertension (91.6%), coronary artery disease (33.3%), atrial fibrillation (25%), chronic obstructive pulmonary disease (25%), diabetes mellitus (16.6%), and autoimmune disorders (16.6%), as shown in (Table 2). At 60-day follow-up, seven patients (58%) were alive, while five (42%) died within 30 days of hospitalization. Non-survivors had a higher median age [83 years, interquartile range (IQR) 80–83] compared with survivors (71 years, IQR 57.5–76.5).

**Table 2:** Baseline demographic and patient’s comorbidities.

Characteristics	Total	Survivor group	Non-survivor group
Number of patients	12	7 (58%)	5 (42%)
Age, median (IQR)	76 (69–83)	71 (57.5–76.5)	83 (80–83)
Male, n (%)	9 (75%)	6 (85.7%)	3 (60%)
Female, n (%)	3 (25%)	1 (14.3%)	2 (20%)
Comorbidities, n (%)			
Arterial hypertension	11 (91.6%)	6 (85.7%)	5 (100%)
Coronary artery disease	4 (33.3%)	2 (28.5%)	2 (40%)
Atrial fibrillation	3 (25%)	2 (28.5%)	1 (20%)
Heart failure	1 (8.3%)	0 (0%)	1 (20%)
Diabetes	2 (16.6%)	0 (0%)	2 (40%)
Obesity ^a^			
Chronic renal disease ^b^	1 (0.8%)	1 (14.2%)	0 (0%)
Chronic liver disease	1 (0.8%)	1 (14.2%)	0 (0%)
Chronic lung disease	3 (25%)	2 (28%)	1 (20%)
Chronic neurological disorder	1 (8.3%)	1 (14.2%)	0 (0%)
Solid malignancy ^c^	1 (8.3%)	1 (14.2%)	0 (0%)
Hematological malignancy ^c^	1 (8.3%)	0 (0%)	1 (20%)
Autoimmune disease	2 (16.6%)	2 (28.5%)	0 (0%)
Organ transplantation	1 (8.3%)	1 (14.2%)	0 (0%)

^a^obesity is defined as body mass index (BMI) > 30 kg/m^2^; ^b^ stage 3–5 of CKD, chronic kidney disease; ^c^ includes solid neoplasia or hematological malignancy in the last 5 years.

CSF profiles were available for all patients (Table 2). Analysis of the initial lumbar puncture revealed pleocytosis with a median leukocyte count of 135 cells/mm^3^ (IQR 79–192), elevated protein levels (median 126 mg/dL, IQR 84–145), and normal glucose concentrations (median 60 mg/dL, IQR 52–71). Most CSF samples demonstrated a lymphocytic predominance. Laboratory findings on admission showed mildly elevated inflammatory markers, with a median white blood cell (WBC) count of 10.6 × 10^3^/mm^3^ (IQR 8.7–12.9), typically accompanied by lymphopenia, and a median C-reactive protein (CRP) level of 3.78 mg/dL (IQR 1.17–8.9). Inflammatory markers were higher among survivors than non-survivors, with median WBC counts of 11.5 × 10^3^/mm^3^ (IQR 8.2–12.4) versus 9.5 × 10^3^/mm^3^ (IQR 8.7–13.5), and CRP levels of 7.04 mg/dL (IQR 2.8–9.3) versus 2.5 mg/dL (IQR 0.76–3.06), respectively (Table 3).

**Table 3:** Laboratory results at hospital admission.

Laboratory markers	Total	Survivor group	Non-survivor group
Number of patients	12	7 (58%)	5 (42%)
CSF profile			
CSF Leukocyte count (cells/mm^3^)	135 (79–192)	90 (79–190)	240 (130–430)
CSF Protein (mg/dL)	126 (84–145)	99 (83.8–145)	163 (136–247)
CSF Glucose (mg/dL)	61 (52–71)	60 (52–71)	62 (59–67)
Blood Test at admission, median (IQR)			
White blood cell count (×10^3^ cells/mm^3^)	10.6 (8.7–12.9)	11.5 (8.2–12.4)	9.5 (8.7–13.5)
Lymphocytes (×10^3^ cells/mm^3^)	0.79 (0.56–0.98)	0.86 (0.64–0.98)	0.75 (0.42–0.94)
Hemoglobin (g/dL)	12.6 (12–13.2)	13 (12.3–13.85)	12.4 (11.8–12.7)
Platelets (×10^3^ cells/mm^3^)	190 (162–263)	185 (151–226)	225 (170–285)
Creatinine (mg/dL)	1.07 (0.73–1.31)	1.04 (0.6–1.18)	1.18 (0.9–1.46)
AST (UI/L)	36 (25–64)	28 (24–46)	47 (32–613)
ALT (UI/L)	31 (23–72)	28 (19.5–35)	67 (25–89)
Total bilirubin (mg/dL)	1.16 (0.96–1.4)	1.22 (0.8–1.5)	1.03 (0.96–1.13)
INR	1.03 (1–1.2)	1.12 (1.01–1.3)	1 (0.98–1.03)
D-Dimer (ng/mL)	880 (780–1,191)	895 (351–1,391)	865 (795–1,170)
C-Reactive Protein (mg/dL)	3.78 (1.17–8.9)	7.04 (2.8–9.3)	2.5 (0.76–3.06)
Procalcitonin (ng/mL)	0.09 (0.05–0.17)	0.125 (0.06–0.17)	0.09 (0.04–0.1)
Diagnostic tests, %			
WNV-specific IgM serum antibodies +	7 (58.3%)	4 (57.1%)	3 (60%)
WNV PCR of CSF +	6 (50%)	4 (57.1%)	2 (40%)
WNV PCR of whole-blood +	8 (66.7%)	5 (71.4%)	3 (60%)
WNV PCR of urine +	5 (41.6%)	3 (42.8%)	2 (40%)

INR, International Normalized Ratio, a standardized measurement of prothrombin time (PT); PCR, polymerase chain reaction.

Regarding the diagnostic work-up, 7 (58.3%) patients had WNV-specific IgM antibodies detected by ELISA in serum, while 8 (66.7%) showed viral RNA by PCR in serum, 6 (50%) in CSF, and 5 (41.6%) in urine.

At admission, the median Glasgow Coma Scale (GCS) score was 12 (IQR 8–13). The median hospital length of stay was 27 days (IQR 17–31). Admission to the ICU occurred after a median of 3 days from hospital presentation (IQR 1.7–3.2), with a median ICU stay of 20 days (IQR 16–21.7). A total of 11 patients (91.6%) required orotracheal intubation and mechanical ventilation, with a median duration of ventilation of 12.5 days (IQR 8.5–17) (Table 4). Overall, five patients (41.7%) died within 30 days of hospitalization, and the 60-day mortality remained unchanged.

**Table 4:** Clinical course of patients during hospital admission.

Clinical characteristics	Median (IQR)	Survivor group	Non-survivor group
Number of patients	12	7 (58%)	5 (42%)
GCS at admission	12 (8–13)	12.5 (7.5–13)	12 (10–13)
Pre-ICU hospitalization, days	3 (1.7–3.2)	3 (2–3.5)	3 (1–3)
Mechanical ventilation, days	12.5 (8.5–17.2)	14 (8–16.5)	11 (10–17)
ICU length of stay, days	20 (16.2–21.7)	20 (20–21)	18 (11–24)
Hospital length of stay, days	27 (17–31)	31 (28.5–35.5)	18 (14–26)
Other characteristics, %			
Brain MRI abnormalities	10 (83%)	6 (85.7%)	4 (80%)
Inotropic/vasopressor support	5 (41.6%)	1 (14.0%)	4 (80%)
Cardiac involvement	4 (33.3%)	1 (14.2%)	3 (60%)
Sepsis complications during hospitalization	3 (25%)	1 (14.2%)	2 (40%)
Outcome			
Orotracheal intubation, no. %	11 (91.6%)	–	–
30-day mortality, no. %	5 (41.7%)	–	–
60-day mortality, no. %	5 (41.7%)	–	–

Brain magnetic resonance imaging (MRI) was performed in all 12 patients. In 2 cases (16.7%), no abnormalities were detected despite the presence of significant neurological symptoms. Five patients (41.6%) required inotropic or vasopressor support during hospitalization, four of whom belonged to the non-survivor group. Three patients (25%) developed sepsis. Cardiac involvement was identified in four patients (33.3%), of which two patients with suspected myocarditis, as diagnosed by consultant cardiologists at each tertiary center based on combined clinical, laboratory, and instrumental findings.

### Case presentations

**Figure 1:**
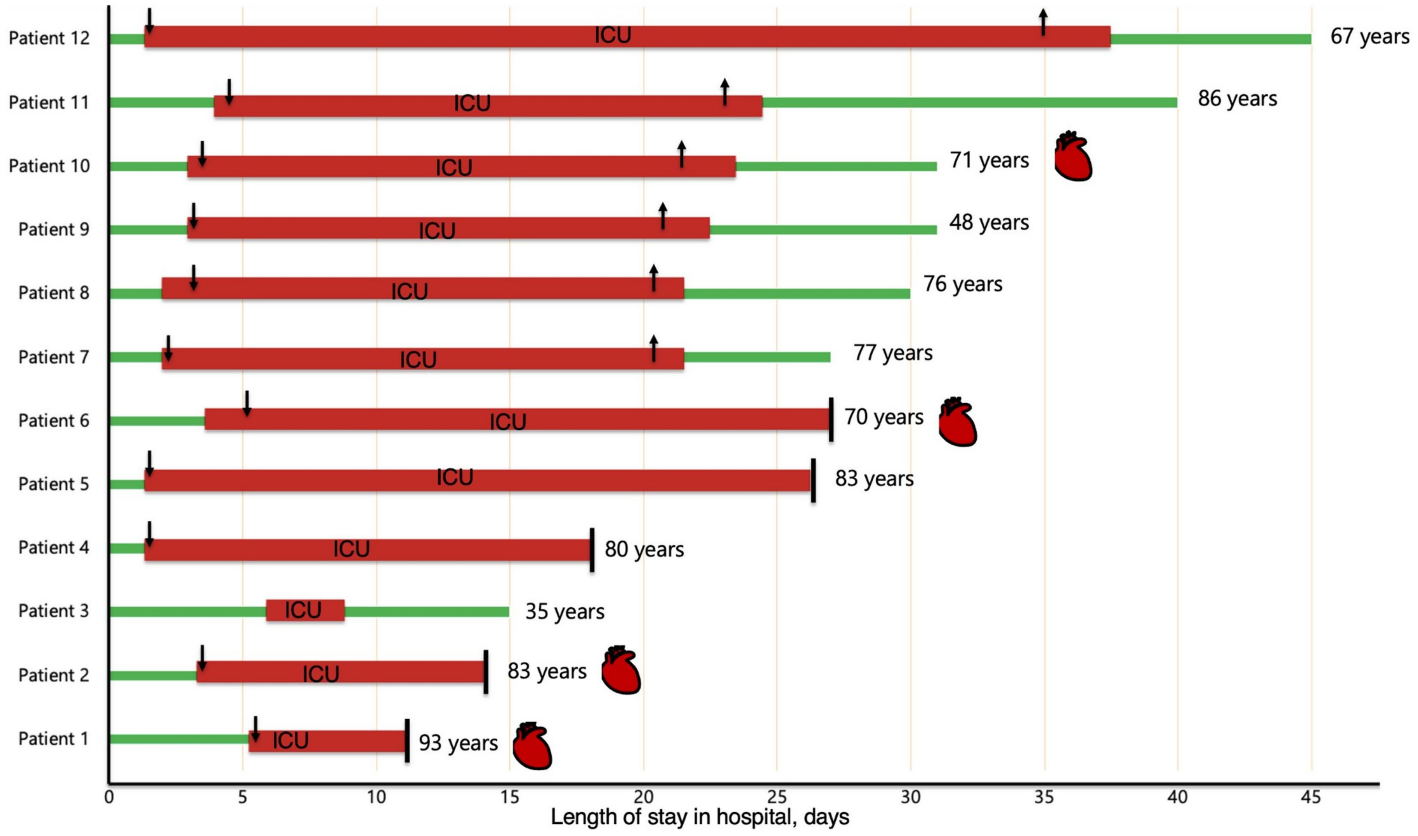
Timeline of patients with neuroinvasive WNV during hospitalization. Green line, ward stay; red line, ICU stay; downward arrow, orotracheal intubation; upward arrow, extubation; vertical bar, exitus; red heart symbol indicates a cardiac involvement with suspected WNV myocarditis.

Figure 1 illustrates the timeline of the 12 WNND patients from hospital admission to discharge or death. Patients 1, 2, 4, 5, and 6 died within 30 days of admission. As depicted in the figure, patients 1, 2, 6, and 10 had cardiac involvement, of which two of them (patient 2 and 6) acute myocardial dysfunction compatible with suspected myocarditis, as confirmed by consultant cardiologists at each tertiary center, as shown in Figure 1 and Table 5. Below, we provide a detailed description of the three most clinically complex cases.

**Table 5:** Clinical, laboratory, electrocardiographic, and echocardiographic features of the four patients with WNV neuroinvasive disease and acute cardiac involvement.

Patient	Gender/Age	Main comorbidities	Hospital presentation	Troponine peak	NTproBNP	Echocardiography findings	ECG alterations	Cardiac involvement	Brain MRI
**1**	F/93y	Hypertension, hypertensive heart disease, type 2 diabetes mellitus, COPD	She presented at hospital with fever, vomiting and confusion	109- > 345- > 765	2,360	LV dysfunction with EF 40%	New onset T-waves inversion in V4-V6	Type II AMI	Hyperintense lesions in long-TR sequences within basal ganglia
**2**	F/83y	Hypertension, type 2 diabetes mellitus	On ED admission she showed neurological decline, after a week of fever, epigastric pain, and diarrhea	100- > 300	1,298	Reduced LV EF (30–35%), diffuse hypokinesia	New onset T-wave inversion in the precordial leads	Suspected myocarditis, Substained ventricular tachycardia, ROSC after ALS	Two foci of hyperintensity on long TR sequences
**6**	M/70y	Hypertension, dyslipidemia, obesity, chronic coronary syndrome with recent PCI and single coronary stent implantation (6 months ago)	He presented at hospital after 9 days of fever, with myalgia, vomiting, and diarrhea. Initial antibiotics were ineffective.	25- > 100- > 350	120- > 4,000	Reduced LV EF (35%) and diffuse hypokinesia, more pronounced in the latero-apical segments, a worsening compared with a previous echocardiography 3 months earlier (EF 45%)	ECG showed new onset biphasic T waves in V3-V6	Suspected myocarditis	T2/FLAIR small areas of hyperintensity on long sequences, without diffusion restriction or post-contrast enhancement, located in the deep periventricular white matter bilaterally in the semioval centers
**10**	M/71y	Chronic lymphocytic leukemia in follow-up, hypertension, active smoker	He presented with flu-like symptoms persisting for 10 days despite antibiotic and corticosteroid therapy. During admission, he developed confusion and dysarthria	25- > 1,917- > 1,235	20,231	Echocardiography performed after the first angiography showed a hypokinesia of the apical segments with apical ballooning appearance, mid-ventricular hypokinesis, hypercontractile basal segments, and severely reduced systolic function (EF 30%). These findings were not limited to the right coronary territory. A previous echocardiogram, performed 12 months earlier, had documented an EF of 50%	ST-segment elevation in V4-V6, DII and aVL	Takotsubo Syndrome, ACS-STEMI treated with PCI-stenting in RCA	Two small hyperintense lesions on long-TR sequences within the left thalamic-capsular region

ED, emergency department; COPD, chronic obstructive pulmonary disease; LV, left ventricular; EF, ejection fraction; ACS, acute coronary syndrome; ECG, electrocardiogram; MRI, magnetic resonance imaging.

### Case 1

An 83-year-old woman with hypertension, diabetes, and hypertensive heart disease presented to the ED with a week of fever, epigastric pain, and diarrhea. She was dehydrated, febrile (38.5 °C), tachycardic (120 bpm), mildly hypoxic (94% SpO2), with elevated creatinine (1.46 mg/dL) and increasing troponin (100–>300 pg./mL). ECG was normal; echocardiography showed global LV hypokinesis [ejection fraction (EF) 40%] without segmental or valvular abnormalities. No recent sick contacts or mosquito bites were reported.

On day two, she developed confusion, limb weakness, and photophobia; CT was unremarkable. Lumbar puncture revealed pleocytosis (245 WBC/mm^3^). WNV IgM was negative, but CSF PCR confirmed WNND. By day three, her GCS dropped to 8–9, and she experienced ventricular tachycardia requiring advanced life support with CPR, electrical cardioversion, followed by return of spontaneous circulation, intubation and ICU admission. A further echocardiography revealed EF decline (30–35%), diffuse hypokinesia, and new ECG T-wave inversions in the precordial leads. Coronary angiography was not performed due to the patient’s critical condition and low pre-test probability for acute coronary syndrome. Based on these findings, acute myocardial dysfunction compatible with suspected myocarditis was diagnosed by the cardiology team. Brain MRI revealed two foci of hyperintensity on long TR sequences, findings attributed to WNV infection.

In ICU, she required inotropic and vasopressor support for refractory hypotension; her condition progressed to cardiogenic shock, resulting in multiorgan failure and death after 11 days.

### Case 2

Case 2 is a 70-year-old man with hypertension, dyslipidemia, and a prior acute coronary syndrome treated with percutaneous coronary intervention (PCI) and placement of a single stent in the left anterior descending artery, with no significant disease in the remaining coronary vessels. He presented after 9 days of fever, myalgia, vomiting, and diarrhea. Initial antibiotics were ineffective. At ED admission, he showed neurological decline with a GCS of 12. Laboratory findings revealed marked systemic inflammation (WBC 13.5 × 10^3^/mm^3^, CRP 35.9 mg/dL), mild troponin increase (25 - > 100 pg./mL), and NT-proBNP (120 pg./mL). ECG showed new biphasic T waves in V3-V6; other vital parameters were stable.

Lumbar puncture indicated CSF pleocytosis (1,100 WBC/mm^3^), with elevated protein (126 mg/dL) and normal glucose. WNV PCR confirmed neuroinvasive disease. After worsening consciousness (GCS 6), on day 5, the patient was intubated and transferred to ICU. Brain MRI showed in T2/FLAIR small areas of hyperintensity on long sequences, without diffusion restriction or post-contrast enhancement, located in the deep periventricular white matter bilaterally in the semioval centers; carotid Doppler was negative for stenosis. Echocardiography revealed newly reduced left ventricular EF (35%) with diffuse hypokinesia, more pronounced in the latero-apical segments, representing worsening compared with an echocardiogram performed 3 months earlier (EF 45%). Cardiac biomarkers further increased (troponin up to 350 pg./mL, NT-proBNP 4,000 pg./mL). Given the low probability of acute coronary syndrome, the cardiologist elected not to perform coronary angiography. In light of the clinical, laboratory and echocardiographic findings as the distribution of wall motion abnormalities was not confined to a single vascular territory, a diagnosis of suspected myocarditis was made by the cardiology team, and the patient was referred for cardiac magnetic resonance imaging (CMR) once his clinical condition stabilized.

During the ICU stay, the patient needed inotropic and vasopressor support and developed acute kidney injury requiring continuous renal replacement therapy (CRRT). The course was further complicated by bloodstream infections with *Acinetobacter baumannii* and *Staphylococcus haemolyticus*, treated with appropriate antibiotics. Despite intensive therapy, he succumbed to refractory septic and cardiogenic shock, resulting in multiorgan failure and death on day 24 of ICU stay.

### Case 3

A 71-year-old man with hypertension, chronic lymphocytic leukemia, and a history of smoking presented with flu-like symptoms persisting for 10 days despite antibiotic and corticosteroid therapy. Initial laboratory tests showed slightly elevated serum creatinine (1.27 mg/dL), high inflammation markers (WBC 14 ×10^3^/mm^3^ and CRP 26.9 mg/dL), normal troponin levels, and markedly elevated NT-proBNP (20,231 pg./mL); chest X-ray indicated perihilar congestion and right basal infiltrates. During the first hospital day, he developed confusion, psychomotor slowing and dysarthria; CSF analysis revealed pleocytosis, and PCR confirmed neuroinvasive WNV. At presentation, GCS was 13, with progressive decline over 48 h. Brain MRI demonstrated two small hyperintense lesions on long-TR sequences within the left thalamic-capsular region, consisting with viral encephalitis. No carotid stenosis was observed in the Doppler ultrasound.

On the third hospital day, neurological deterioration required ICU transfer and intubation. ECG later showed ST-segment elevation in V4-V6, DII and aVL, with a rise in high-sensitivity troponin T (25- > 1,917- > 1,235 pg./mL). He underwent urgent coronary angiography which showed right coronary artery stenosis treated by angioplasty and stent implantation, while other vessels were clear. Two days after angioplasty, owing to persistent ST-segment elevation in the lateral leads, the patient underwent a repeat coronary angiography, which demonstrated stent patency and no new lesions in the remaining coronary vessels.

Transthoracic echocardiography performed after the first angiography showed a hypokinesia of the apical segments with apical ballooning appearance, mid-ventricular hypokinesis, hypercontractile basal segments, and severely reduced systolic function (EF 30%). These findings were not limited to the right coronary territory. A previous echocardiogram, performed 12 months earlier, had documented an EF of 50%. Takostubo syndrome was established based on echocardiographic findings and widespread ventricular repolarization abnormalities on ECG, neither of which correlated with the isolated single-vessel coronary stenosis found on angiography. Consequently, the patient was referred for cardiac magnetic resonance imaging (CMR), upon clinical stabilization to further investigate the possibility of acute myocardial dysfunction superimposed on ischemic heart disease.

During the ICU stay, the patients required norepinephrine support for the first 10 days. In the meantime, he started on started on dual antiplatelets therapy (DAPT) and empiric antibiotic treatment for suspected pneumonia found on chest CT, although bronchoalveolar lavage (BAL) was negative, and gas exchange remained satisfactory under mechanical ventilation. After 1 week, he developed thrombocytopenia was found (platelet count 30 × 10^3^/μL); however, due to the high ischemic risk, DAPT was continued. After 15 days in ICU, gradual weaning from MV was initiated. During weaning, he was alert, cooperative, and able to move all four limbs, maintaining ad adequate spontaneous respiratory drive. On day 17, he was successfully extubated. Nevertheless, he continued to experience episodes of psychomotor agitation alternating with psychomotor slowing. On day 20 in ICU, he was transferred from the ICU to the medical ward.

After 31 days of hospitalization, he continued to have residual lower-limb weakness and was transferred to a cardiorespiratory and motor rehabilitation center, with progressive improvement. Cardiac MRI for was planned but not performed post-discharge due to claustrophobia. At 3 months of follow-up, he achieved complete neurological recovery, and repeat echocardiography showed improvement of LF ejection fraction (EF 40–45%), without signs of congestive heart failure and NT-proBNP fell to 540 pg./mL (from initially 20,231 pg./mL).

## Discussion

**Figure 2:**
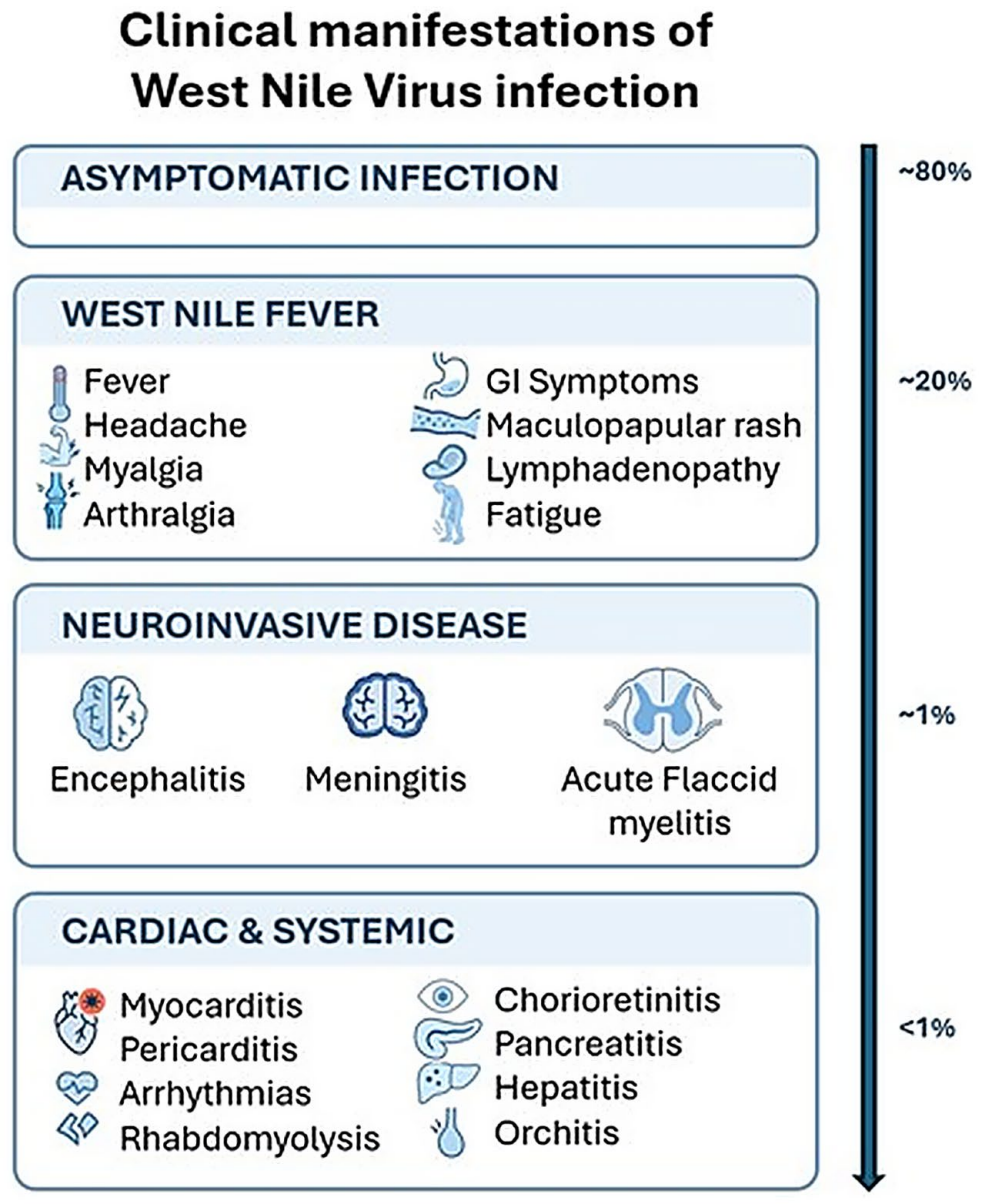
A diagram showing the clinical presentation of WNV from mild to severe cases.

This multicenter case series describes the clinical spectrum, diagnostic features, and outcomes of 12 critically ill patients with West Nile neuroinvasive disease (WNND) admitted to intensive care units during the 2025 outbreak in Central Italy. Among 197 patients with confirmed WNV infection, 64 (32%) developed neuroinvasive disease, and 12 (6.1%) required ICU admission, reflecting a severe clinical phenotype within a large regional outbreak. Overall 30-day mortality reached 41.7%, underscoring the substantial burden of critical illness associated with WNND. As shown in Figure 2, the clinical presentation of WNV infection varies according to current literature. The demographic profile of our ICU cohort was consistent with previously reported series, with advanced age and a high prevalence of cardiovascular and metabolic comorbidities (19–21). Non-survivors were older and had a greater burden of chronic disease. However, given the limited sample size, these findings should be interpreted as descriptive observations rather than independent predictors. The high rate of mechanical ventilation (91.6%) and prolonged ICU stay (median 20 days) emphasize the significant resource utilization and prolonged organ support frequently required in severe WNND, in line with earlier ICU-focused report (9, 22–24).

**Figure 3:**
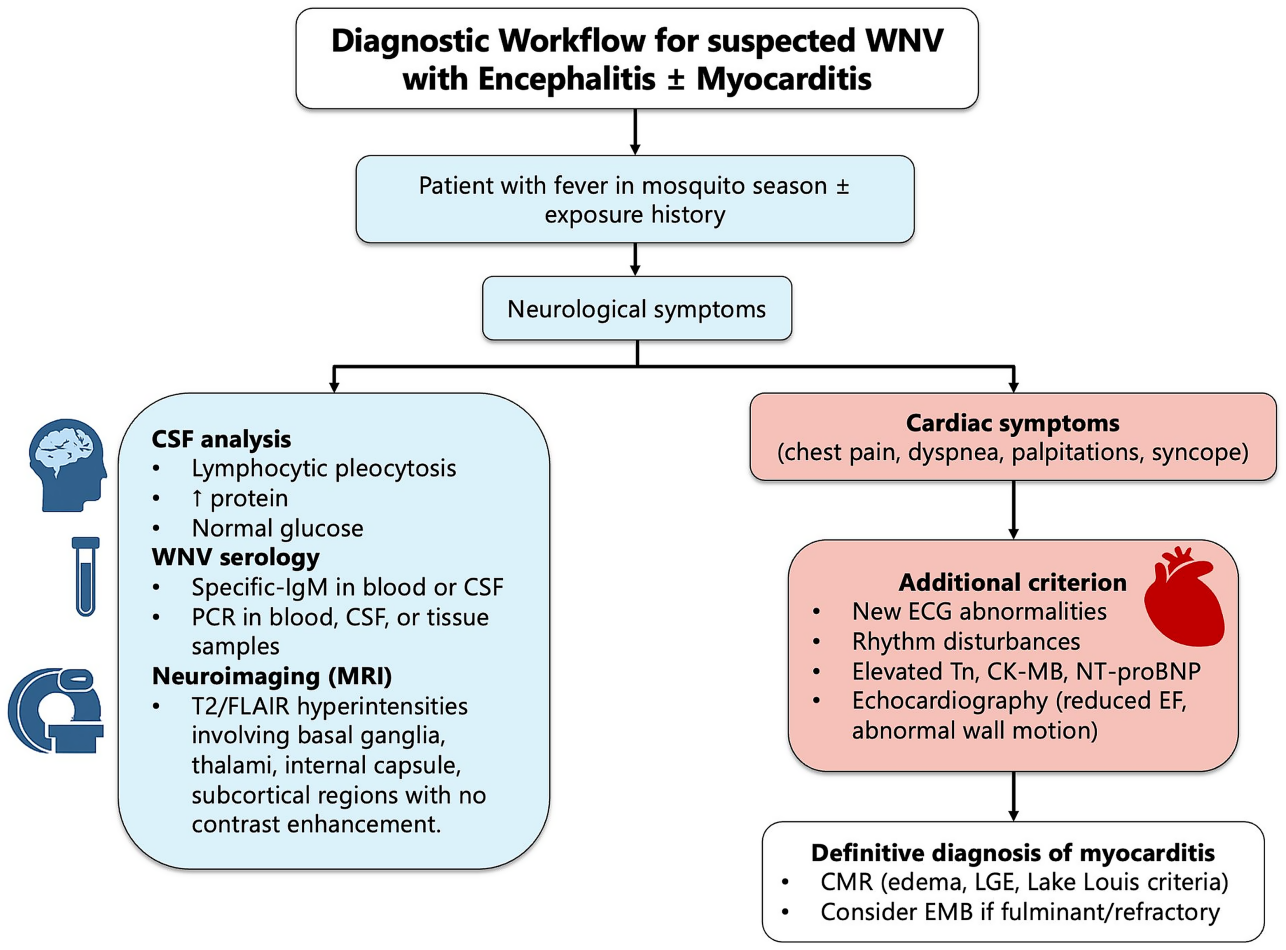
A diagram showing the diagnostic workflow for WNV encephalitis with or without cardiac involvement. CK-MB, Creatin kinase MB; NT-proBNP, N-terminal pro B-type natriuretic peptide; EF, Ejection fraction; CMR, Cardiac magnetic resonance; EMB, Endomyocardial biopsy.

**Figure 4:**
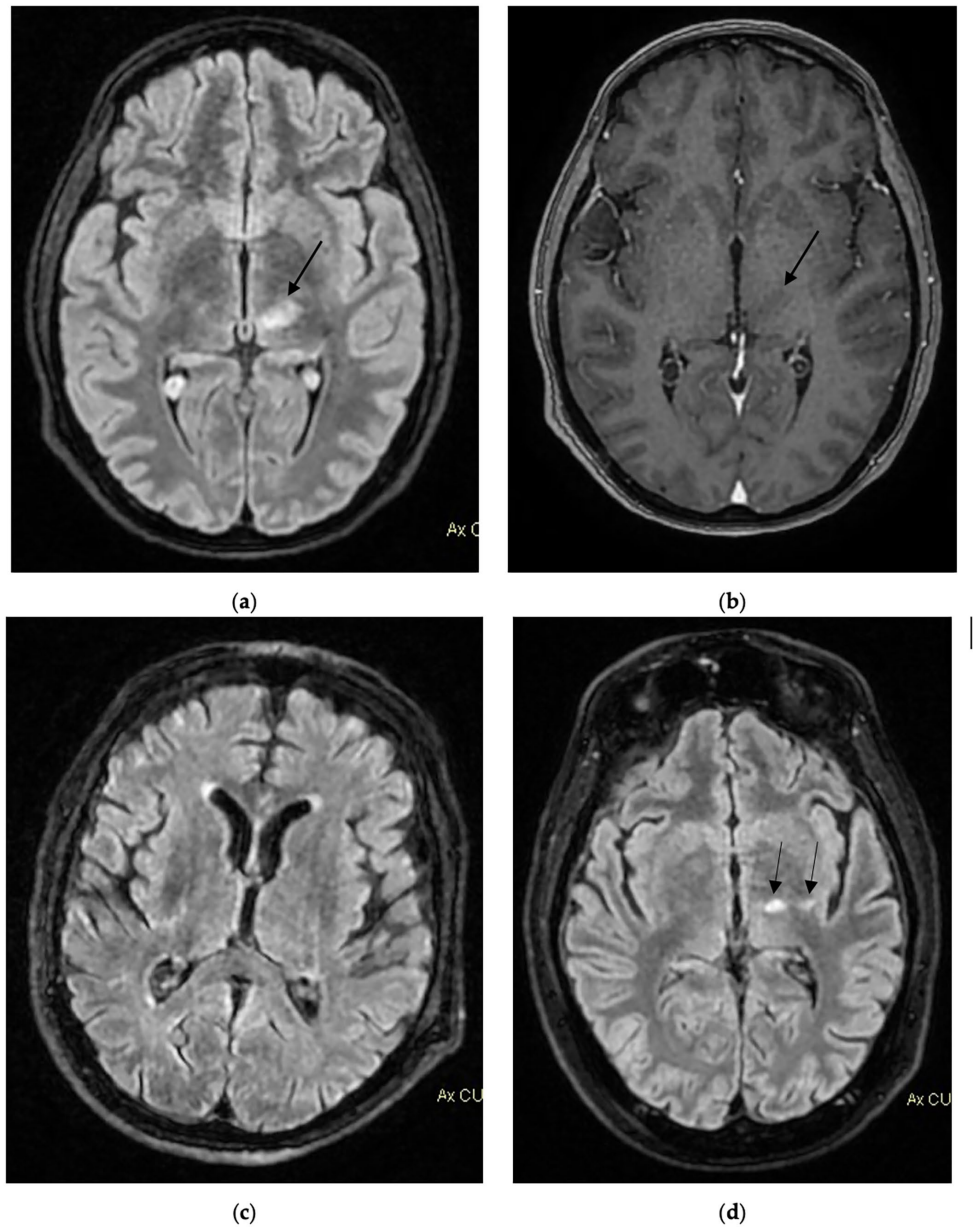
Representative brain MRI images from selected cases: Case 1, two foci of hyperintensity on long TR sequences **(a)** and axial T1 gradient echo contrast-enhanced **(b)**—one in the left thalamus (14 × 8 mm), showing no contrast enhancement; Case 2, **(c)** Axial T2/FLAIR image demonstrating small areas of hyperintensity on long TR sequences, located in the deep periventricular white matter bilaterally and in the corona radiata (semioval centers); Case 3, **(d)** Axial T2/FLAIR image showing two subcentimetric pseudonodular lesions in the left thalamus and internal capsule region, appearing hyperintense on long TR sequences.

An interesting descriptive finding was the higher WBC and CRP levels at admission among survivors. Although the small sample size precludes definitive conclusions, one possible interpretation is that a more pronounced early inflammatory response may reflect preserved host immune reactivity, whereas a blunted response could be associated with immune dysfunction and poorer outcomes. Alternatively, differences in timing of presentation, bacterial co-infection, or other confounding variables may partially explain this observation. These findings should therefore be considered hypothesis-generating rather than causal. Similar findings have been described in prior WNV cohorts, where a blunted inflammatory response correlated with severe outcome (25, 26). Diagnosis of WNV infection relies on combined serological and molecular testing, as both the window of viremia and the timing of antibody response vary across individuals (27). An example of diagnostic workflow for suspected WNV infection is shown in Figure 3. Neurological presentation followed the typical biphasic pattern described in the literature, with initial flu-like or gastrointestinal symptoms preceding progressive encephalopathy, limb weakness, and decreased level of consciousness (25, 28) Cerebrospinal fluid findings were consistent with viral meningoencephalitis, showing lymphocytic pleocytosis, elevated protein, and preserved glucose levels. Brain MRI was performed in all 12 patients, predominantly involving the thalami and brainstem (Figure 4), consistent with previously reported radiologic patterns (29, 30). In two cases, no significative abnormalities were detected despite significant neurological impairment, highlighting that a normal MRI does not exclude neuroinvasive disease (31).

Importantly, normal MRI findings in two patients confirm that neuroimaging may be non-diagnostic in the early or diffuse stages of WNND. A notable finding of this series is the frequency of cardiac involvement, observed in four of 12 ICU patients (33.3%), although, given the inherent limitations of a case series design, these findings should be interpreted strictly as descriptive observations.

Two patients fulfilled criteria for suspected myocarditis, while one was diagnosed with Takotsubo syndrome and one with type II myocardial infarction in the context of critical illness. All patients with cardiac involvement developed new left ventricular systolic dysfunction accompanied by biomarker elevation and electrocardiographic abnormalities. Three of the four patients with cardiac involvement died, suggesting a possible association between myocardial dysfunction and poor outcome in this critically ill cohort.

Cardiac manifestations of WNV infection have been described mainly in isolated case reports and small series and pathophysiological mechanism include both cardiomyocyte invasion and immune-mediated injury (32–34).

Importantly, systematic cardiac evaluation was performed only in ICU patients, and therefore the true prevalence of cardiac involvement among the broader WNV population remains uncertain. Nevertheless, the high frequency observed in this critically ill cohort suggests that myocardial dysfunction may be under-recognized in severe WNND and warrants increased clinical awareness.

From a management perspective, the absence of specific antiviral therapy for WNV infection means that supportive care remains the cornerstone of treatment (13, 19, 35, 36).

In our cohort, management required close collaboration between intensivists, neurologists, infectious disease specialists, and cardiologists. Early identification of neurological deterioration and prompt hemodynamic and respiratory support were essential components of care.

Given the potential for myocardial involvement, systematic cardiac assessment—including ECG monitoring, serial troponin measurements, and echocardiography—may be considered in patients with severe WNND, particularly when hemodynamic instability or arrhythmias occur.

### Limitations

This study has several limitations. First, the sample size was small and derived from two tertiary centers, reason why no statistic tests are reported. The findings should therefore be interpreted as descriptive rather than inferential. Second, the patients did not undergo the same standardized cardiac work-up (serial biomarkers, repeated echo, ECG monitoring), because a specific study protocol was not pre-set. Furthermore, the diagnosis of myocarditis was not histologically confirmed, as endomyocardial biopsy was deemed unsafe in critically ill patients. Similarly, CMR could not be performed because of hemodynamic instability or contraindications, potentially leading to under- or over-diagnosis of cardiac involvement. Third, virological and immunological characterization (e.g., viral load quantification, genomic sequencing, cytokine profiling) was not available, preventing a deeper understanding of pathophysiological mechanisms or viral strain virulence.

Finally, long-term follow-up was limited, and post-discharge cardiac and neurological recovery could only be documented in a subset of survivors. Fourth, due to the observational design and variability in laboratory sampling intervals, a systematic longitudinal analysis of inflammatory marker trajectories was not feasible.

Despite these limitations, this series provides valuable real-world insight into the severe, multisystemic presentation of WNV neuroinvasive disease requiring ICU admission, highlighting the importance of integrated neuro-cardiac evaluation and supportive critical-care management.

## Conclusion

In summary, this case series highlights the severe multisystemic nature of WNND requiring intensive care, characterized by high mortality, prolonged organ support, and a substantial burden of cardiac complications. While the findings remain descriptive, they underscore the importance of integrated neuro-cardiac evaluation and provide a foundation for future prospective studies aimed at clarifying the mechanisms, prevalence, and prognostic impact of WNV-related myocardial injury.

## Data Availability

The original contributions presented in the study are included in the article/supplementary material, further inquiries can be directed to the corresponding author.
